# Covariance Eigenspace Provides Latent Attribution of Longitudinal Effects in Brain Age Gap

**Published:** 2026-10-15

**Authors:** Jason Scheffel, Saurabh Sihag

## Abstract

Brain age gap is a promising machine learning (ML)-driven bio\-marker of accelerated biological aging and is derived from neuroimaging data. Recent works on coVariance neural networks (VNN) for brain age gap prediction have provided an explainable and anatomically verifiable framework for brain age gap prediction. Notably, VNN outcomes are derived by filtering inputs along covariance eigenvectors, providing a mechanism for explaining their decisions in terms of latent (orthogonal) vectors. In this paper, we investigate the longitudinal brain age gap effects in a Mild Cognitive Impairment (MCI) cohort using a VNN-driven brain age gap prediction framework. Specifically, using Integrated Gradients (IG) as the attribution mechanism, our results demonstrate that the eigenspectrum of the anatomical covariance matrix explains the different longitudinal effects observed in brain age gap in amyloid positive vs amyloid negative subcohorts more prominently than individual brain regions ($83.2\%$ with the leading eigenvector versus a maximal value of $7.7\%$ for any brain region). Therefore, the covariance-based latent explanation identifies a key coordinated input pattern that carries most of the observed longitudinal effect in brain age gap.

